# Supplementary material for: Performance of family health teams for tackling chronic diseases in a state of the Amazon
Source: PLoS One. 2020 Nov 6;15(11):e0241765. doi: 10.1371/journal.pone.0241765 (PMC7647065; doi:10.1371/journal.pone.0241765)
Supplement: S1 Table — PMAQ-AB Cycle 2, Tocantins, Northern Brazil. Definitions of abbreviations: CI = confidence interval; PMAQ-AB = primary care access and quality improvement program; NASF = family health support center; NCDs = noncommunicable diseases; SIAB = primary care information system; e-SUS = Unified Health System electronic information; COPD = chronic obstructive pulmonary disease; PHUs = primary health units. (DOCX) [file pone.0241765.s001.docx]

**S1 Table. Descriptive analysis of the work process variables of family health teams (n = 361) used in the study. PMAQ-AB Cycle 2, Tocantins, Northern Brazil.**

| **Variables** | **n** | **%(95%CI)** |
| --- | --- | --- |
| Participates in Continuing Education^a^ | 310 | 86.8(82.9– 90.0) |
| Participates in Continuing Education through Telehealth | 126 | 34.9(30.2–40.0) |
| Acts in PHUs that receives students, teachers and / or researchers | 167 | 46.3(41.2–51.4) |
| Performs monthly planning with a document proving | 246 | 68.1(63.2–72.7) |
| Performs indicator monitoring | 333 | 92.2(89.0–94.6) |
| Participated in PMAQ-AB cycle 1 | 250 | 69.3(64.3–73.8) |
| Receives matrix support from the NASF to care for people with NCDs | 147 | 40.7(35.8–45.9) |
| Receives matrix support from the Health Academy pole^b^ | 71 | 21.4(17.3–26.1) |
| Has maps updated for at least 1 year of coverage area | 187 | 51.8(46.7–56.9) |
| Has an information system (SIAB or e-SUS) for recording information^c^ | 333 | 94.9(92.0–96.7) |
| Stores electronic medical records on computer | 38 | 10.5(7.8–14.1) |
| Performs triage of hypertensive crisis and hyperglycemia in diabetics | 338 | 93.6(90.6–95.7) |
| Works at PHUs with appointment scheduling any day of the week and time | 191 | 52.9(47.8–58.0) |
| Works at PHUs with appointment by phone | 92 | 25.5(21.3–30.2) |
| Performs risk assessment and vulnerability in the triage and were trained | 225 | 62.3(57.2–67.2) |
| Provides user removal service when needed | 332 | 92.0(88.7–94.4) |
| Offers services to women’s groups | 316 | 87.5(83.7–90.6) |
| Offers services to tobacco user group | 129 | 35.7(31.0–40.8) |
| Offers services to group of users of alcohol and other drugs | 140 | 38.8(33.9–43.9) |
| Offers services to group of users with obesity | 161 | 44.6(39.6–49.8) |
| Offers services to group of elderly users | 326 | 90.3(86.8–93.0) |
| Offers services to group of users with hypertension | 335 | 92.8(89.7–95.0) |
| Offers services to group of users with hypertension | 333 | 92.2(89.0–94.6) |
| Offers services to group of users with COPD | 133 | 36.8(32.0–41.9) |
| Performs recipe renewal for users with hypertension and diabetes without appointment booking | 317 | 87.8(84.0–90.8) |
| Acts in PHUs that immediately schedule expert consultation for users | 70 | 19.4(15.6–23.8) |
| Acts in PHUs that later schedule expert consultation for users | 189 | 52.4(47.2–57.5) |
| Has a record of women eligible for cervical cytopathological screening | 252 | 69.8(64.9–74.3) |
| Has record of women eligible for mammogram | 111 | 30.7(26.2–35.7) |
| Has records of users with hypertension | 319 | 88.4(84.7–91.3) |
| Has records of users with diabetes | 316 | 87.5(83.7–90.6) |
| Has record of users with COPD | 61 | 16.9(13.4–21.1) |
| Has records of users with obesity | 61 | 16.9(13.4–21.1) |
| Offers consultations for users with hypertension | 354 | 98.1(96.1–99.1) |
| Offers consultations for users with diabetes | 353 | 97.8(95.7–98.9) |
| Offers consultations for users with obesity | 169 | 46.8(41.7–52.0) |
| Offers consultation for users with COPD | 148 | 41.0(36.0–46.1) |
| Uses protocols for cervical cancer risk stratification | 200 | 55.4(50.2–60.5) |
| Conducts active search for cases of delayed cervical cancer screening | 228 | 63.2(58.1–68.0) |
| Uses protocols for breast cancer risk stratification | 161 | 44.6(39.6–49.8) |
| Uses protocols for hypertension risk stratification | 184 | 51.0(45.8–56.1) |
| Uses protocols for diabetes risk stratification | 184 | 51.0(45.8–56.1) |
| Uses protocols for COPD risk stratification | 101 | 28.0(23.6–32.8) |
| Performs active search for cases of cervical cancer | 243 | 67.3(62.3–72.0) |
| Performs active search for cases of breast cancer | 181 | 50.1(45.0–55.3) |
| Performs active search for cases of hypertension | 265 | 73.4(68.6–77.7) |
| Performs active search for cases of diabetes | 259 | 71.7(66.9–76.1) |
| Performs active search for cases of alcohol and drug use | 85 | 23.5(19.5–28.2) |
| Requests creatinine test performed by the service network | 354 | 98.1(96.1–99.1) |
| Requests lipid profile test performed by the service network | 345 | 95.6(92.9–97.3) |
| Requests electrocardiogram performed by the service network | 327 | 90.6(87.1–93.2) |
| Requests electrocardiogram performed by the service network | 277 | 76.7(72.1–80.8) |
| Requests glycosylated hemoglobin test performed by the service network | 330 | 91.4(88.1–93.9) |
| Requests mammogram performed by the service network | 337 | 93.4(90.3–95.5) |
| Requests fasting glucose test performed by the service network | 359 | 99.4(98.0–99.9) |
| Works at PHUs that collect blood test | 153 | 42.4(37.4–47.5) |
| Works at PHUs that collect urine test | 149 | 41.3(36.3–46.4) |
| Works at the PHUs that collect cytopathological cervix screening | 316 | 87.5(83.7–90.6) |
| Works at PHUs that perform electrocardiogram exam | 15 | 4.2(2.5–6.7) |
| Works at PHUs that perform nebulization / inhalation | 332 | 92.0(88.7–94.4) |
| Always get feedback from experts reviews from referred users | 54 | 15.0(11.7–19.0) |
| Referred users wait up to 30 days for cardiologist consultation^d^ | 173 | 50.1(44.9–55.4) |
| Referred users wait up to 30 days for ophthalmologist consultation^e^ | 170 | 50.9(45.6–56.2) |
| Referred users wait up to 30 days for mammography exam^f^ | 246 | 71.9(66.9–76.4) |
| Performs and records weight and height of users with hypertension and diabetes | 340 | 94.2(91.3–96.2) |
| Engages the NASF to support the monitoring of obese users in PHUs | 158 | 43.8(38.7–48.9) |
| Offers educational and health promotion actions for healthy eating^c^ | 280 | 79.8(75.3–83.6) |
| Offers educational and health promotion actions to self-management support for NCDs^c^ | 229 | 63.4(60.1–70.0 |
| Offers educational and health promotion actions addressing alcohol, crack and other drugs^c^ | 142 | 40.5(35.5–45.7 |
| Encourages and develops physical activity^c^ | 247 | 70.4(65.4–74.9 |
| Records families registered in the Bolsa Familia Program with document proving | 239 | 66.2(61.2–70.9 |
| Provides home care for users in need that care | 346 | 95.8(93.3–97.5 |
| Performs user satisfaction assessment | 192 | 53.2(48.0–58.3 |
| Acts in area with Local Health Council or popular participation spaces with document proving | 118 | 32.7(28.1–37.7 |
| Performs activities in schools for early detection of hypertension^g^ | 194 | 59.1(53.8–64.3 |
| Performs activities in schools for nutritional assessment^g^ | 265 | 80.8(76.2–84.7 |
| Performs activities in schools to promote healthy eating^g^ | 278 | 84.8(80.5–88.2 |
| Performs activities in schools to promote physical activity^g^ | 208 | 63.4(58.1–68.4 |
| Performs activities in schools to train teachers for health education^g^ | 119 | 36.3(31.26–41.6 |
| Asks all users about tobacco use | 150 | 41.6(36.6–46.7 |
