# Supplementary material for: Performance of family health teams for tackling chronic diseases in a state of the Amazon
Source: PLoS One. 2020 Nov 6;15(11):e0241765. doi: 10.1371/journal.pone.0241765 (PMC7647065; doi:10.1371/journal.pone.0241765)
Supplement: S2 Table — PMAQ-AB Cycle 2, Tocantins, Northern Brazil. Definitions of abbreviations: CI = confidence interval; PMAQ-AB = primary care access and quality improvement program; NASF = family health support center; NCDs = noncommunicable diseases; SIAB = primary care information system; e-SUS = Unified Health System electronic information; COPD = chronic obstructive pulmonary disease; PHUs = primary health units. Definitions of symbols: † = Factor loading ≥ 0.4. (DOCX) [file pone.0241765.s002.docx]

**S2 Table. Factor loading of variables of work process of family health teams captured by the CATPCA method. PMAQ-AB Cycle 2, Tocantins, Northern Brazil.**

| **Variables** | **Principal components** | | | | | |
| --- | --- | --- | --- | --- | --- | --- |
|  | **1** | **2** | **3** | **4** | **5** | **6** |
| Participates in Continuing Education | 0.202 | 0.111 | 0.325 | 0.017 | -0.077 | 0.196 |
| Participates in Continuing Education through Telehealth | 0.333 | -0.062 | 0.109 | -0.092 | 0.258 | 0.017 |
| Acts in PHUs that receives students, teachers and / or researchers | 0.196 | 0.173 | 0.244 | -0.227 | -0.145 | 0.177 |
| Performs monthly planning with a document proving | 0.340 | -0.049 | 0.200 | -0.219 | -0.012 | -0.012 |
| Performs indicator monitoring | 0.319 | -0.097 | 0.225 | -0.071 | -0.106 | -0.054 |
| Participated in PMAQ-AB cycle 1 | 0.097 | 0.017 | 0.148 | -0.021 | -0.118 | 0.111 |
| Receives matrix support from the NASF to care for people with NCDs | 0.120 | -0.131 | 0.305 | **0.507**† | -0.085 | -0.128 |
| Receives matrix support from the Health Academy pole | 0.168 | -0.064 | 0.176 | 0.018 | 0.015 | 0.051 |
| Has maps updated for at least 1 year of coverage area | 0.267 | 0.050 | 0.146 | -0.326 | -0.052 | 0.195 |
| Has an information system (SIAB or e-SUS) for recording information | -0.049 | -0.026 | 0.063 | -0.196 | -0.011 | 0.166 |
| Stores electronic medical records on computer | 0.278 | 0.177 | -0.030 | **-0.430**† | -0.067 | 0.111 |
| Performs triage of hypertensive crisis and hyperglycemia in diabetics | 0.123 | -0.034 | 0.194 | -0.095 | 0.016 | 0.067 |
| Works at PHUs with appointment scheduling any day of the week and time | 0.256 | -0.257 | 0.027 | -0.088 | -0.078 | 0.163 |
| Works at PHUs with appointment by phone | -0.007 | -0.252 | 0.058 | 0.101 | 0.094 | 0.020 |
| Performs risk assessment and vulnerability in the triage and were trained | 0.392 | -0.062 | 0.047 | -0.067 | 0.078 | 0.146 |
| Provides user removal service when needed | 0.185 | -0.035 | 0.104 | -0.060 | 0.177 | 0.014 |
| Offers services to women’s groups | 0.353 | **-0.575**† | -0.033 | -0.015 | -0.184 | -0.007 |
| Offers services to tobacco user group | 0.365 | -0.078 | -0.114 | 0.242 | -0.203 | 0.091 |
| Offers services to group of users of alcohol and other drugs | **0.421**† | -0.246 | -0.171 | 0.256 | -0.221 | 0.004 |
| Offers services to group of users with obesity | **0.523**† | -0.296 | -0.061 | 0.225 | -0.149 | -0.118 |
| Offers services to group of elderly users | 0.375 | **-0.516**† | -0.158 | 0.069 | -0.099 | 0.055 |
| Offers services to group of users with hypertension | 0.371 | **-0.659**† | -0.076 | -0.037 | -0.203 | 0.059 |
| Offers services to group of users with hypertension | 0.378 | **-0.657**† | -0.086 | -0.032 | -0.202 | 0.064 |
| Offers services to group of users with COPD | **0.466**† | -0.206 | -0.162 | 0.185 | -0.264 | -0.029 |
| Performs recipe renewal for users with hypertension and diabetes without appointment booking | 0.252 | 0.041 | -0.132 | -0.077 | 0.079 | -0.085 |
| Acts in PHUs that immediately schedule expert consultation for users | 0.181 | -0.036 | 0.031 | -0.253 | -0.258 | 0.023 |
| Acts in PHUs that later schedule expert consultation for users | 0.162 | 0.020 | 0.032 | -0.011 | -0.076 | 0.354 |
| Has a record of women eligible for cervical cytopathological screening | 0.191 | 0.155 | -0.011 | -0.339 | 0.218 | 0.107 |
| Has record of women eligible for mammogram | **0.414**† | 0.167 | -0.163 | -0.122 | 0.156 | 0.168 |
| Has records of users with hypertension | 0.386 | -0.045 | -0.184 | -0.302 | 0.007 | -0.025 |
| Has records of users with diabetes | 0.374 | -0.051 | -0.197 | -0.307 | -0.013 | -0.020 |
| Has record of users with COPD | **0.449**† | 0.100 | -0.077 | -0.126 | 0.067 | 0.091 |
| Has records of users with obesity | **0.539**† | 0.078 | -0.090 | -0.109 | 0.024 | 0.047 |
| Offers consultations for users with hypertension | 0.128 | -0.112 | -0.034 | -0.322 | -0.285 | 0.380 |
| Offers consultations for users with diabetes | 0.129 | -0.163 | -0.032 | -0.323 | -0.309 | **0.418**† |
| Offers consultations for users with obesity | **0.530**† | -0.143 | -0.001 | 0.171 | -0.179 | 0.124 |
| Offers consultation for users with COPD | **0.490**† | -0.173 | -0.028 | 0.214 | -0.153 | 0.105 |
| Uses protocols for cervical cancer risk stratification | **0.656**† | 0.103 | 0.075 | -0.174 | 0.279 | -0.301 |
| Conducts active search for cases of delayed cervical cancer screening | **0.413**† | **0.420**† | -0.381 | 0.133 | -0.129 | -0.058 |
| Uses protocols for breast cancer risk stratification | **0.666**† | 0.070 | 0.037 | -0.139 | 0.258 | -0.268 |
| Uses protocols for hypertension risk stratification | **0.658**† | 0.105 | 0.054 | -0.197 | 0.213 | -0.365 |
| Uses protocols for diabetes risk stratification | **0.657**† | 0.108 | 0.043 | -0.206 | 0.202 | -0.360 |
| Uses protocols for COPD risk stratification | **0.595**† | 0.069 | 0.073 | -0.125 | 0.127 | -0.120 |
| Performs active search for cases of cervical cancer | **0.415**† | **0.465**† | -0.370 | 0.213 | -0.169 | -0.024 |
| Performs active search for cases of breast cancer | **0.486**† | 0.300 | -0.345 | 0.202 | -0.098 | -0.017 |
| Performs active search for cases of hypertension | **0.437**† | 0.505 | -0.343 | 0.176 | -0.276 | -0.043 |
| Performs active search for cases of diabetes | **0.448**† | 0.523 | -0.308 | 0.181 | -0.289 | -0.058 |
| Performs active search for cases of alcohol and drug use | **0.487**† | 0.098 | -0.230 | 0.234 | -0.114 | 0.121 |
| Requests creatinine test performed by the service network | 0.178 | 0.102 | **0.520**† | -0.077 | -0.242 | -0.241 |
| Requests lipid profile test performed by the service network | 0.176 | 0.153 | **0.445**† | -0.082 | -0.199 | -0.198 |
| Requests electrocardiogram performed by the service network | 0.217 | 0.274 | **0.489**† | -0.001 | -0.294 | -0.128 |
| Requests electrocardiogram performed by the service network | 0.190 | 0.122 | 0.367 | 0.019 | -0.235 | -0.106 |
| Requests glycosylated hemoglobin test performed by the service network | 0.213 | 0.162 | **0.511**† | 0.030 | -0.189 | -0.036 |
| Requests mammogram performed by the service network | 0.162 | 0.117 | **0.545**† | 0.050 | -0.318 | -0.071 |
| Requests fasting glucose test performed by the service network | 0.119 | 0.117 | **0.464**† | -0.042 | -0.102 | -0.136 |
| Works at PHUs that collect blood test | -0.013 | 0.352 | 0.170 | 0.191 | 0.051 | **0.648**† |
| Works at PHUs that collect urine test | -0.017 | 0.355 | 0.171 | 0.160 | 0.034 | **0.633**† |
| Works at the PHUs that collect cytopathological cervix screening | 0.078 | 0.134 | 0.269 | 0.075 | 0.030 | 0.316 |
| Works at PHUs that perform electrocardiogram exam | 0.100 | -0.018 | 0.058 | -0.088 | 0.118 | 0.257 |
| Works at PHUs that perform nebulization / inhalation | 0.215 | -0.180 | -0.096 | -0.119 | -0.064 | 0.153 |
| Always get feedback from experts reviews from referred users | -0.005 | -0.108 | -0.024 | 0.013 | -0.034 | 0.036 |
| Referred users wait up to 30 days for cardiologist consultation | 0.099 | -0.167 | 0.098 | 0.310 | 0.163 | 0.062 |
| Referred users wait up to 30 days for ophthalmologist consultation | 0.117 | -0.104 | 0.050 | 0.222 | 0.233 | 0.056 |
| Referred users wait up to 30 days for mammography exam | 0.263 | -0.039 | 0.016 | 0.147 | 0.189 | 0.046 |
| Performs and records weight and height of users with hypertension and diabetes | 0.087 | -0.128 | 0.123 | -0.128 | -0.009 | 0.068 |
| Engages the NASF to support the monitoring of obese users in PHUs | 0.249 | -0.062 | 0.288 | **0.460**† | -0.135 | -0.115 |
| Offers educational and health promotion actions for healthy eating^c^ | 0.251 | 0.258 | -0.114 | 0.049 | 0.182 | 0.175 |
| Offers educational and health promotion actions to self-management support for NCDs | 0.337 | 0.119 | -0.058 | 0.065 | 0.106 | 0.142 |
| Offers educational and health promotion actions addressing alcohol, crack and other drugs | 0.308 | 0.058 | -0.153 | 0.144 | 0.071 | 0.101 |
| Encourages and develops physical activity | 0.252 | -0.028 | 0.039 | 0.229 | **0.401**† | 0.234 |
| Records families registered in the Bolsa Familia Program with document proving | 0.338 | -0.059 | -0.083 | -0.184 | 0.356 | 0.067 |
| Provides home care for users in need that care | 0.140 | -0.021 | -0.025 | -0.086 | -0.026 | 0.160 |
| Performs user satisfaction assessment | 0.338 | -0.033 | 0.254 | -0.274 | 0.140 | 0.212 |
| Acts in area with Local Health Council or popular participation spaces with document proving | 0.084 | -0.244 | -0.055 | 0.055 | 0.333 | 0.017 |
| Performs activities in schools for early detection of hypertension | 0.250 | -0.075 | 0.187 | 0.201 | 0.219 | 0.067 |
| Performs activities in schools for nutritional assessment | 0.191 | -0.037 | 0.268 | 0.343 | 0.202 | 0.071 |
| Performs activities in schools to promote healthy eating | 0.182 | -0.078 | 0.380 | 0.226 | 0.296 | 0.114 |
| Performs activities in schools to promote physical activity | 0.270 | -0.037 | 0.155 | 0.308 | **0.400**† | 0.079 |
| Performs activities in schools to train teachers for health education | 0.357 | -0.087 | 0.092 | 0.063 | 0.250 | -0.045 |
| Asks all users about tobacco use | **0.400**† | 0.009 | 0.046 | 0.112 | 0.087 | 0.054 |
| **Eigenvalue** | **8.731** | **3.859** | **3.697** | **3.041** | **2.751** | **2.675** |
| **Variance (%)** | **10.91** | **4.82** | **4.62** | **3.80** | **3.44** | **3.34** |
| **Cronbach’s alpha** | **0.897** | **0.750** | **0.739** | **0.680** | **0.644** | **0.634** |
| **Scores median** | **-0.0167** | **-0.0690** | **0.0841** | **-0.0155** | **0.0516** | **0.0071** |
