# Supplementary material for: Performance of family health teams for tackling chronic diseases in a state of the Amazon
Source: PLoS One. 2020 Nov 6;15(11):e0241765. doi: 10.1371/journal.pone.0241765 (PMC7647065; doi:10.1371/journal.pone.0241765)
Supplement: S3 Table — Definitions of abbreviations: COPD = chronic obstructive pulmonary disease; NASF = Family Health Support Center; NCDs = noncommunicable diseases; PHUs = primary health units. (DOCX) [file pone.0241765.s003.docx]

**S3 Table. Pairwise comparison analysis of the differences between the health regions, Tocantins, Northern Brazil.**

| **Variables** | |
| --- | --- |
| Offers services to group of users of alcohol and other drugs | |
| pair | p-value |
| 1 vs. 2 | < 0.001 |
| 1 vs. 3 | 0.004 |
| 1 vs. 7 | 0.038 |
| 2 vs. 5 | < 0.001 |
| 2 vs. 6 | 0.019 |
| 2 vs. 7 | 0.035 |
| Offers services to group of users with obesity | |
| pair | p-value |
| 1 vs. 2 | < 0.001 |
| 1 vs. 7 | 0.014 |
| 2 vs. 3 | 0.007 |
| 2 vs. 5 | 0.004 |
| 2 vs. 6 | 0.043 |
| Offers services to group of users with COPD | |
| pair | p-value |
| 1 vs. 2 | 0.002 |
| 2 vs; 4 | 0.010 |
| 2 vs. 5 | < 0.001 |
| 2 vs. 6 | 0.022 |
| 2 vs.7 | 0.006 |
| Has record of women eligible for mammogram | |
| pair | p-value |
| 1 vs. 4 | 0.049 |
| 1 vs. 5 | < 0.001 |
| 1 vs. 7 | < 0.001 |
| 2 vs. 5 | < 0.001 |
| 2 vs. 7 | < 0.001 |
| 3 vs. 5 | 0.004 |
| 3 vs. 7 | 0.007 |
| Offers consultations for users with obesity | |
| pair | p-value |
| 1 vs. 4 | 0.002 |
| 1 vs. 5 | 0.025 |
| 1 vs. 6 | 0.021 |
| 2 vs. 4 | < 0.001 |
| 2 vs. 5 | < 0.001 |
| 2 vs. 6 | 0.026 |
| 2 vs. 7 | 0.041 |
| 3 vs. 4 | < 0.001 |
| 3 vs. 5 | < 0.001 |
| 4 vs. 6 | 0.009 |
| 4 vs. 7 | < 0.001 |
| 4 vs. 8 | 0.002 |
| 5 vs. 7 | 0.005 |
| 5 vs. 8 | 0.014 |
| Offers consultations for users with COPD | |
| pair | p-value |
| 1 vs. 2 | < 0.001 |
| 1 vs. 5 | 0.034 |
| 2 vs. 4 | < 0.001 |
| 2 vs. 5 | < 0.001 |
| 2 vs. 6 | 0.005 |
| 2 vs 7 | 0.001 |
| 3 vs 4 | < 0.001 |
| 3 vs 5 | 0.011 |
| 4 vs 7 | 0.027 |
| 4 vs 8 | 0.009 |
| Uses protocols for cervical cancer risk stratification | |
| pair | p-value |
| 1 vs. 2 | < 0.001 |
| 1 vs. 3 | < 0.001 |
| 1 vs. 5 | 0.028 |
| 1 vs. 8 | 0.046 |
| 2 vs. 4 | < 0.001 |
| 2 vs. 5 | < 0.001 |
| 2 vs. 7 | 0.001 |
| 3 vs. 4 | 0.001 |
| 3 vs. 5 | < 0.001 |
| 3 vs. 7 | 0.016 |
| 4 vs. 5 | 0.025 |
| 5 vs. 6 | < 0.001 |
| 5 vs. 7 | < 0.001 |
| 5 vs. 8 | < 0.001 |
| Performs active search for cases of delayed cervical cancer screening | |
| pair | p-value |
| 1 vs. 7 | < 0.001 |
| 2 vs. 3 | 0.006 |
| 2 vs. 7 | 0.046 |
| 3 vs. 4 | 0.021 |
| 3 vs. 5 | 0.004 |
| 3 vs. 7 | < 0.001 |
| 3 vs. 8 | 0.033 |
| 4 vs. 7 | 0.006 |
| 5 vs. 7 | 0.024 |
| 6 vs. 7 | < 0.001 |
| Uses protocols for breast cancer risk stratification | |
| pair | p-value |
| 1 vs. 2 | < 0.001 |
| 1 vs. 3 | 0.003 |
| 2 vs. 4 | < 0.001 |
| 2 vs. 5 | < 0.001 |
| 2 vs. 6 | 0.033 |
| 3 vs. 4 | < 0.001 |
| 3 vs. 5 | < 0.001 |
| 4 vs. 7 | 0.013 |
| 4 vs. 8 | 0.015 |
| 5 vs. 6 | 0.015 |
| 5 vs. 7 | < 0.001 |
| 5 vs. 8 | 0.002 |
| Uses protocols for hypertension risk stratification | |
| pair | p-value |
| 1 vs. 2 | < 0.001 |
| 1 vs. 3 | < 0.001 |
| 1 vs. 8 | 0.016 |
| 2 vs. 7 | 0.049 |
| 3 vs. 4 | 0.034 |
| 3 vs. 5 | < 0.001 |
| 3 vs. 7 | 0.024 |
| 4 vs. 5 | 0.002 |
| 5 vs. 6 | 0.001 |
| 5 vs. 7 | < 0.001 |
| 5 vs. 8 | < 0.001 |
| Uses protocols for diabetes risk stratification | |
| pair | p-value |
| 1 vs. 2 | < 0.001 |
| 1 vs. 3 | < 0.001 |
| 1 vs. 5 | 0.026 |
| 2 vs. 4 | 0.035 |
| 2 vs. 5 | < 0.001 |
| 2 vs. 7 | 0.049 |
| 3 vs. 4 | 0.018 |
| 3 vs. 5 | < 0.001 |
| 3 vs. 6 | 0.046 |
| 3 vs. 7 | 0.024 |
| 4 vs. 5 | 0.002 |
| 5 vs. 6 | 0.002 |
| 5 vs. 7 | < 0.001 |
| 5 vs. 8 | < 0.001 |
| Uses protocols for COPD risk stratification | |
| pair | p-value |
| 1 vs. 2 | 0.004 |
| 1 vs. 3 | 0.012 |
| 1 vs. 5 | 0.023 |
| 1 vs. 8 | 0.037 |
| 2 vs. 4 | 0.023 |
| 2 vs. 5 | < 0.001 |
| 3 vs. 5 | < 0.001 |
| 4 vs. 5 | 0.017 |
| 5 vs. 6 | 0.004 |
| 5 vs. 7 | 0.001 |
| 5 vs. 8 | < 0.001 |
| Performs active search for cases of cervical cancer | |
| pair | p-value |
| 1 vs. 2 | 0.032 |
| 1 vs. 5 | 0.021 |
| 1 vs. 7 | < 0.001 |
| 2 vs. 3 | < 0.001 |
| 2 vs. 6 | 0.001 |
| 2 vs. 7 | 0.031 |
| 3 vs. 4 | 0.027 |
| 3 vs. 5 | <0.001 |
| 3 vs. 7 | <0.001 |
| 4 vs. 6 | 0.031 |
| 4 vs. 7 | 0.005 |
| 5 vs. 6 | < 0.001 |
| 5 vs. 7 | 0.022 |
| 6 vs. 7 | < 0.001 |
| 7 vs. 8 | 0.010 |
| Performs active search for cases of breast cancer | |
| pair | p-value |
| 3 vs. 4 | 0.006 |
| 3 vs. 5 | 0.002 |
| 3 vs. 7 | 0.001 |
| 6 vs. 7 | 0.041 |
| Performs active search for cases of hypertension | |
| pair | p-value |
| 1 vs. 3 | < 0.001 |
| 1 vs. 7 | 0.028 |
| 2 vs. 3 | < 0.001 |
| 2 vs. 6 | 0.019 |
| 3 vs. 4 | 0.004 |
| 3 vs. 5 | < 0.001 |
| 3 vs. 7 | < 0.001 |
| 3 vs. 8 | 0.030 |
| 4 vs. 7 | 0.046 |
| 5 vs. 6 | 0.046 |
| 5 vs. 7 | 0.034 |
| 6 vs. 7 | < 0.001 |
| 7 vs. 8 | 0.039 |
| Performs active search for cases of diabetes | |
| pair | p-value |
| 1 vs. 3 | 0.002 |
| 1 vs. 7 | < 0.001 |
| 2 vs. 3 | < 0.001 |
| 2 vs. 6 | 0.003 |
| 3 vs. 4 | 0.017 |
| 3 vs. 5 | < 0.001 |
| 3 vs. 7 | < 0.001 |
| 3 vs. 8 | 0.046 |
| 4 vs. 7 | 0.005 |
| 5 vs. 6 | 0.009 |
| 5 vs. 7 | 0.034 |
| 6 vs. 7 | < 0.001 |
| 7 vs. 8 | 0.039 |
| Performs active search for cases of alcohol and drug use | |
| pair | p-value |
| 3 vs. 4 | 0.001 |
| 3 vs. 5 | 0.009 |
| 3 vs. 7 | 0.014 |
| Asks all users about tobacco use | |
| pair | p-value |
| 1 vs. 7 | 0.017 |
| 2 vs. 5 | 0.002 |
| 3 vs. 5 | < 0.001 |
| 4 vs. 7 | 0.019 |
| 5 vs. 6 | 0.014 |
| 5 vs. 7 | < 0.001 |
| 5 vs. 8 | 0.008 |
| Offers services to women’s groups | |
| pair | p-value |
| 1 vs. 2 | < 0.001 |
| 1 vs. 8 | < 0.001 |
| 2 vs. 3 | 0.009 |
| 2 vs. 4 | < 0.001 |
| 2 vs. 5 | < 0.001 |
| 2 vs. 6 | < 0.001 |
| 2 vs. 7 | < 0.001 |
| 3 vs. 8 | 0.003 |
| 4 vs. 8 | 0.004 |
| 5 vs. 8 | 0.014 |
| 6 vs. 8 | 0.001 |
| 7 vs. 8 | 0.016 |
| Has record of users with COPD | |
| pair | p-value |
| 1 vs. 4 | 0.020 |
| 1 vs. 5 | < 0.001 |
| 1 vs. 7 | 0.009 |
| 1 vs. 8 | 0.004 |
| 2 vs. 5 | 0.016 |
| 3 vs. 5 | 0.041 |
| 4 vs. 8 | 0.041 |
| 5 vs. 8 | 0.005 |
| 7 vs. 9 | 0.027 |
| Has record of users with obesity | |
| pair | p-value |
| 1 vs. 4 | 0.020 |
| 1 vs. 5 | < 0.001 |
| 1 vs. 7 | 0.009 |
| 1 vs. 8 | 0.004 |
| 2 vs. 5 | 0.004 |
| 3 vs. 5 | 0.006 |
| Offers services to group of elderly users | |
| pair | p-value |
| 1 vs. 2 | < 0.001 |
| 1 vs. 8 | 0.034 |
| 2 vs. 3 | 0.002 |
| 2 vs. 4 | < 0.001 |
| 2 vs. 5 | < 0.001 |
| 2 vs. 6 | < 0.001 |
| 2 vs. 7 | 0.002 |
| 4 vs. 8 | 0.037 |
| 6 vs. 8 | 0.016 |
| Offers services to group of users with hypertension | |
| pair | p-value |
| 1 vs. 2 | < 0.001 |
| 1 vs. 8 | 0.014 |
| 2 vs. 3 | < 0.001 |
| 2 vs. 4 | < 0.001 |
| 2 vs. 5 | < 0.001 |
| 2 vs. 6 | < 0.001 |
| 2 vs. 7 | < 0.001 |
| 3 vs. 8 | 0.036 |
| 4 vs. 8 | 0.009 |
| 5 vs. 8 | 0.006 |
| 6 vs. 8 | 0.016 |
| 7 vs. 8 | 0.036 |
| Offers services to group of users with diabetes | |
| pair | p-value |
| 1 vs. 2 | < 0.001 |
| 1 vs. 8 | 0.034 |
| 2 vs. 3 | < 0.001 |
| 2 vs. 4 | < 0.001 |
| 2 vs. 5 | < 0.001 |
| 2 vs. 6 | < 0.001 |
| 2 vs. 7 | < 0.001 |
| 3 vs. 8 | 0.036 |
| 4 vs. 8 | 0.009 |
| 5 vs. 8 | 0.006 |
| 6 vs. 8 | 0.016 |
| 7 vs. 8 | 0.036 |
| Requests electrocardiogram performed by the service network | |
| pair | p-value |
| 1 vs. 3 | < 0.001 |
| 2 vs. 3 | 0.002 |
| 3 vs. 4 | < 0.001 |
| 3 vs. 5 | < 0.001 |
| 3 vs. 6 | 0.032 |
| 3 vs. 7 | 0.029 |
| 3 vs. 8 | 0.030 |
| 4 vs. 7 | 0.039 |
| Requests glycosylated hemoglobin test performed by the service network | |
| pair | p-value |
| 4 vs. 8 | 0.037 |
| 5 vs. 7 | 0.017 |
| 5 vs. 8 | 0.015 |
| Solicita glicemia | |
| pair | p-value |
| 1 vs. 3 | < 0.001 |
| 1 vs. 6 | 0.046 |
| 3 vs. 4 | 0.004 |
| 3 vs. 5 | 0.004 |
| Receives matrix support from the NASF to care for people with NCDs | |
| pair | p-value |
| 1 vs. 2 | < 0.001 |
| 1 vs. 3 | < 0.001 |
| 1 vs. 5 | < 0.001 |
| 1 vs. 7 | 0.002 |
| 1 vs. 8 | 0.006 |
| 2 vs. 4 | < 0.001 |
| 2 vs. 5 | < 0.001 |
| 3 vs. 4 | < 0.001 |
| 3 vs. 5 | < 0.001 |
| 4 vs. 5 | < 0.001 |
| 4 vs. 6 | 0.036 |
| 4 vs. 7 | 0.001 |
| 4 vs. 8 | 0.004 |
| 5 vs. 6 | < 0.001 |
| 5 vs. 8 | 0.001 |
| Stores electronic medical records on computer | |
| pair | p-value |
| 1 vs. 5 | < 0.001 |
| 2 vs. 5 | < 0.001 |
| 3 vs. 6 | < 0.001 |
| 4 vs. 5 | < 0.001 |
| 5 vs. 6 | < 0.001 |
| Engages the NASF to support the monitoring of obese users in PHUs | |
| pair | p-value |
| 1 vs. 3 | 0.002 |
| 1 vs. 5 | < 0.001 |
| 1 vs. 7 | < 0.001 |
| 1 vs. 8 | 0.002 |
| 2 vs. 4 | 0.039 |
| 2 vs. 5 | 0.002 |
| 3 vs. 4 | < 0.001 |
| 4 vs. 5 | < 0.001 |
| 4 vs. 7 | < 0.001 |
| 4 vs. 8 | < 0.001 |
| 5 vs. 6 | 0.014 |
| Encourages and develops physical activity | |
| pair | p-value |
| 1 vs. 6 | < 0.001 |
| 1 vs. 7 | 0.014 |
| 2 vs. 6 | 0.001 |
| 3 vs. 6 | < 0.001 |
| 3 vs. 7 | 0.017 |
| 4 vs. 7 | 0.012 |
| 4 vs. 6 | < 0.001 |
| 5 vs. 6 | < 0.001 |
| 6 vs. 7 | 0.019 |
| Works at PHUs that collect blood test | |
| pair | p-value |
| 1 vs. 2 | < 0.001 |
| 1 vs. 4 | 0.019 |
| 2 vs. 3 | < 0.001 |
| 2 vs. 5 | 0.009 |
| 2 vs. 6 | < 0.001 |
| 2 vs. 7 | < 0.001 |
| 2 vs. 8 | < 0.001 |
| 3 vs. 4 | 0.013 |
| 4 vs. 6 | 0.009 |
| 4 vs. 8 | 0.009 |
| 5 vs. 6 | 0.033 |
| 5 vs. 8 | 0.029 |
| Works at PHUs that collect urine test | |
| pair | p-value |
| 1 vs. 2 | < 0.001 |
| 1 vs. 4 | 0.019 |
| 1 vs. 5 | 0.036 |
| 2 vs. 3 | < 0.001 |
| 2 vs. 5 | 0.021 |
| 2 vs. 6 | < 0.001 |
| 2 vs. 7 | < 0.001 |
| 2 vs. 8 | < 0.001 |
| 3 vs. 4 | 0.004 |
| 4 vs. 6 | 0.009 |
| 4 vs. 7 | 0.008 |
| 4 vs. 8 | 0.009 |
| 5 vs. 6 | 0.017 |
| 5 vs. 7 | 0.014 |
