# Supplementary material for: Performance of family health teams for tackling chronic diseases in a state of the Amazon
Source: PLoS One. 2020 Nov 6;15(11):e0241765. doi: 10.1371/journal.pone.0241765 (PMC7647065; doi:10.1371/journal.pone.0241765)
Supplement: Attachment — Submitted filename: Response to Reviewers.docx [file pone.0241765.s004.docx]

PONE-D-19-35881
**Performance of the family health teams for tackling chronic diseases in a state of the Amazon.**
PLOS ONE

**RESPONSE TO EDITOR AND REVIEWERS**

**RESPONSE TO EDITOR:**

**Comment:**

Dear Dr. Gomes Alves,

Thank you for submitting your manuscript to PLOS ONE. After careful consideration, we feel that it has merit but does not fully meet PLOS ONE’s publication criteria as it currently stands. Therefore, we invite you to submit a revised version of the manuscript that addresses the points raised during the review process.

Please address the changes recommended by the reviewers. In addition, please address the following:

**Response from the authors:** Thank you for your careful review and comments on our manuscript submitted to PlosOne. We are submitting the manuscript which was reviewed according to reviewers' comments and recommendations. In addition, this letter presents the responses of every note made by the reviewers. The changes made to the manuscript were highlighted in red. We certify that our article meets the standards of the journal.

**Comment:** Page 13: Lines 110-111 - Please clarify the following statement: “The state is divided into eight health regions (Fig. 1) that were instituted after meeting the following inclusion criteria. I would assume that the health regions exist despite the study, so not sure how the inclusion criteria were used to develop the 8 regions? Page 14: Lines 130-131 - Can you describe these 3 phases? Page 15: Line 144- logical should be changed to logic I believe.

**Response from the authors:** Dear Editor, we are grateful for your careful review of our manuscript. We made all the necessary changes which are in red and answered the questions raised. Then, we explained how was divided the 8 health regions in the Tocantins, we described the 3 PMAQ-AB phases and we correct all terms from logical to logic.

**Comment:** As noted by both reviewers, I would also strongly recommend that you include more information on the strategy itself as well as a description of the Family Health Teams.

Furthermore, I would ask that you carefully review the manuscript for minor edits such as spelling, use of words, and other errors.

**Response from the authors:** Dear Editor, we are grateful for your comment. We made all the recommended changes and we added more information about the Family Health Strategy and the family health teams. The revised manuscript was submitted on AJE (American Journal Experts) for English review.

**RESPONSE TO REVIEWERS**

**Comment - Reviewer #1:** This is an important evaluation of the performance of the Brazilian primary care in light of the Chronic Care Model and recent national reforms aimed at controlling non-communicable diseases. The study utilized existing data and subjected it to a robust analysis. A minor limitation in the report is a lack of detail in describing the "Family Health Strategy" and the "Family Health Teams". These are the focus of the research and warrant more detailed description since these are not generic to other health systems. Currently the discussion is of a higher order and does not adequately describe these well enough for readers not familiar with the Brazilian primary care reforms. The principal components which constitute the data gives the reader a sense of what Family Health Teams do. However, other details such as the level of training (cadre) about these health care providers are lacking. It would be worth citing literature about Family Health Teams.
A few typographical errors are present in the manuscript and would need attention through further proofreading.

**Response from the authors:** Dear reviewer, we are grateful for your careful review of our manuscript. We added more information about the Family Health Strategy and family health teams in the Introduction. We added in the Result section the information that the variables used in this study are part of the set of health actions expected for family health teams to perform according to the Family Health Strategy policy. We believe that information clarified the Discussion about the performance of the teams, what they do and what they would have to do. Studies that evaluate the outcomes of the Family Health Strategy on chronic disease health indicators are incipient in Brazil and our study is innovative. Unfortunately, variables about the level of training of family health teams were not used because of missing data. But, it is known that most teams received training on family medicine (for physicians) and family health (for nurses, dentists, and other NASF professionals non-physicians). We made the discussion more clear to the readers and we added more information about other Family Health Strategy research.

**Comment - Reviewer #2:** The aim of the study was to evaluate the adequacy of the work process among family health teams and to compare regional adequacy differences in the state of Tocantins, in Amazonian Region, Brazil. A cross-sectional study was conducted to evaluate the adequacy of the work process among family health teams and compare regional adequacy differences in the state of Tocantins, in Amazonian Region, Brazil. It was carried out a cross-sectional using the PMAQ-AB secondary database. The method is described with rigor and details. The results and discussions answered adequately the study’s aim. The methodology evaluation of the family health teams for tackling NCDs is innovative in the Family Health Strategy, in Brazil. It was possible to determine six PC associated with the family health teams' work process adequacy in the Tocantins health regions and to compare the findings among these regions.
My recommendation for the authors is to include in the introduction more information related to the work process and the composition of the family health teams. It is important because the variables selected from the PMAQ-AB national database concerned the family health teams' work process. In addition, it is important to highlight the specifics of work process and family health teams in primary care in Brazil.

**Response from the authors:** Dear reviewer, we are grateful for your careful review of our manuscript. We added more information about Family Health Strategy and family health teams in the Introduction. We added in the Result section the information that the variables used in this study are part of the set of health actions expected for family health teams to perform according to the Family Health Strategy policy. We believe that information clarified the Discussion about the performance of the teams, what they do and what they would have to do. In the Discussion section, we also clarified what the family health teams really are doing in a Primary Health Care through Family Heath Strategy.
