# Supplementary material for: Performance of family health teams for tackling chronic diseases in a state of the Amazon
Source: PLoS One. 2020 Nov 6;15(11):e0241765. doi: 10.1371/journal.pone.0241765 (PMC7647065; doi:10.1371/journal.pone.0241765)
Supplement: Attachment — Submitted filename: Response to Reviewers.docx [file pone.0241765.s005.docx]

PONE-D-19-35881_R1
**Performance of the family health teams for tackling chronic diseases in a state of the Amazon.**
PLOS ONE

**RESPONSE TO EDITOR**

**RESPONSE TO EDITOR:**

**Comment:**

Dear Dr. Gomes Alves,

Thank you for submitting your manuscript to PLOS ONE. After careful consideration, we feel that it has merit but does not fully meet PLOS ONE’s publication criteria as it currently stands. Therefore, we invite you to submit a revised version of the manuscript that addresses the points raised during the review process.

Please attend to the minor revisions requested. All revisions are required for consideration of this manuscript for publication.

**Response from the authors:** Thank you for your careful review and comments on our manuscript submitted to PlosOne. We are submitting the manuscript which was reviewed according to Editor’ comments and recommendations. In addition, this letter presents the responses of every note made by the Editor. The manuscript was revised with track changes. We certify that our article meets the standards of the journal. In addition, the manuscript was revised in English by the American Journal Experts.

**Comment:** Final sentence in the abstract is not clear. As it currently stands, it does not make sense. Please revise. Would also suggest breaking up into two sentences.

**Response from the authors:** Thank you for your careful review. We broke up the final sentence into two sentences to make it more clear.

**Comment:** Line 67, delete “in order.” Not necessary.

**Response from the authors:** Thank you for your comment. We deleted “in order”.

**Comment:** Lines 80-81, “reduce the population's health indicators” is not clear.

**Response from the authors:** Thank you for your comment. We rewrote the sentence to make it clearer.

**Comment:** Line 117, remove the first “the.”

**Response from the authors:** Thank you for your comment. We removed the first “the”.

**Comment:** Line 137, suggest capitalizing “Indigenous.”

**Response from the authors:** Thank you for your suggestion. We capitalize “Indigenous”.

**Comment:** Line 169, “adherence to family health,” change the “to” to “of”

**Response from the authors:** Thank you for your comment. We made that change.

**Comment:** Line 210: “other NASF professionals nonphysicians,” this is not clear. Also need to spell out acronym.

**Response from the authors:** Thank you for your comment. We rewrote the sentence to make it clearer.

**Comment:** Review manuscript for consistency in capitalization of “Regions” when referring to a specific numbered region. They should all be capitalized.

**Response from the authors:** Thank you for your comment. We made all the capitalized to refer to specific “Region”.
